# Intestinal microbiota score could predict survival following allogeneic hematopoietic stem cell transplantation

**DOI:** 10.1007/s00277-022-04817-8

**Published:** 2022-03-25

**Authors:** Lijie Han, Haiyan Zhang, Ping Ma, Jie Peng, Yilu Li, Jiaying Wu, Yuanyuan Li, Jifeng Yu, Wei Li, Mengmeng Zhang, Jia bao He, Zhiping Fan, Weimin Wang, Li’na Sang, Hui Sun, Qifa Liu, Yang Liu, Zhongxing Jiang

**Affiliations:** 1 Department of Hematology, The First Affiliated Hospital of Zhengzhou University, Zhengzhou, China; 2 Department of Hematology, Nanfang Hospital, Southern Medical University, Guangzhou, China; 3 Hematology/Oncology Department, Children’s Hospital Affiliated To Zhengzhou University, Zhengzhou, China; 4 Department of Oncology, the Second Affiliated Hospital of Guizhou Medical University, Kaili, China; 5 School of Foreign Languages, Henan University of Chinese Medicine, Zhengzhou, China; 6 Guangdong Provincial Key Laboratory of Construction and Detection in Tissue Engineering, Southern Medical University, Guangzhou, China; 7 Department of Radiotherapy, Affiliated Cancer Hospital of Zhengzhou University, Henan Cancer Hospital, Zhengzhou, China

**Keywords:** Allogeneic, Hematopoietic stem cell transplantation, Intestinal microbiota, Survival

## Abstract

Intestinal microbiota is an important prognostic factor for allogeneic hematopoietic stem cell transplantation (allo-HSCT), but its role in predicting survival has not been determined. Here, stool samples at day 15 ± 1 posttransplant were obtained from 209 patients at two centers. Microbiota was examined using 16S rRNA sequencing. The microbiota diversity and abundance of specific bacteria (including Lachnospiraceae, Ruminococcaceae, Erysipelotrichaceae, and Enterobacteriaceae) were assigned a value of 0 or 1 depending on whether they were positive or negative associated with survival, respectively. An accumulated intestinal microbiota (AIM) score was generated, and patients were divided into low- and high-score groups. A low score was associated with a better 3-year cumulative overall survival (OS) as well as lower mortality than a high score (88.5 vs. 43.9% and 7.1 vs. 35.8%, respectively; both *P* < 0.001). In multivariate analysis, a high score was found to be an independent risk factor for OS and transplant-related mortality (hazard ratio = 5.68 and 3.92, respectively; *P* < 0.001 and 0.003, respectively). Furthermore, the AIM score could serve as a predictor for survival (area under receiver operating characteristic curve = 0.836, *P* < 0.001). Therefore, the intestinal microbiota score at neutrophil recovery could predict survival following allo-HSCT.

**Supplementary Information:**

The online version contains supplementary material available at 10.1007/s00277-022-04817-8.

## Introduction

Allogeneic hematopoietic stem cell transplantation (allo-HSCT) is a curative option for hematological malignancies. Complications such as graft-versus-host disease (GVHD) and infections remain major causes of death beyond malignancy relapse, limiting the application of allo-HSCT [1, 2].

Increasing studies have demonstrated that the intestinal microbiome plays an important role in host physiology during allo-HSCT [3–5]. Previous studies have indicated that patients undergoing allo-HSCT easily suffer from microbiota injury owing to the use of antibiotic agents and conditioning [3, 4, 6]. We and others have reported that the microbiota disruption caused by allo-HSCT is characterized by expansions in pathogenic bacteria and loss of diversity, a variable that reflects the number of unique bacterial taxa present and their relative frequencies [4, 5, 7]. Diversity of the intestinal microbiota has previously been correlated with GVHD, infection, relapse, and toxic effects on organs. Importantly, loss of diversity is associated with transplant- and GVHD-related mortality [3, 4, 8]. Recently, a study has shown that intestinal microbiota diversity, which is associated with mortality, is a biomarker for predicting survival. However, it is unclear which bacteria are closely associated with survival. For example, a study indicated that Enterococcus expansion was associated with GVHD and mortality [9], while another study showed that loss of Blautia was associated with mortality [10]. Therefore, a divergence of the relationships exists between microbiota and outcomes of allo-HSCT, and identifying the bacteria that could predict survival is essential.

Furthermore, it is necessary to confirm whether these bacteria together could serve as prognostic predictors for survival. In this study, we prospectively obtained stool samples from patients undergoing allo-HSCT on day 15 ± 1 posttransplantation. The relationship between microbiota and survival was further estimated, and a microbiota score derived from the combination of diversity and several specific bacteria was developed, which could suitably predict survival after allo-HSCT.

## Methods

### Subjects and samples

We performed a retrospective analysis of a prospective study recruiting allo-HSCT recipients from the Nanfang Hospital and The First Affiliated Hospital of Zhengzhou University between January 2016 and December 2018. Stool samples were collected from patients at approximately day 15 ± 1 posttransplantation. The samples were labeled and stored at − 80 °C until DNA extraction [4, 7]. If the period between collection and disposition was longer than 6 h, the samples were discarded. This study was approved by the ethics committee of the Nanfang Hospital, Southern Medical University, and The First Affiliated Hospital of Zhengzhou University, China. After approval of the study by the ethics committee, consent from the participants was obtained for biospecimen collection and analysis. This study was conducted in accordance with the Declaration of Helsinki.

### Conditioning and GVHD prophylaxis

The conditioning regimens used in this study included two standard myeloablative regimens (busulfan + cyclophosphamide (BuCY) and total body irradiation + CY (TBI + CY)) and a sequential intensified regimen (fludarabine + Ara-C plus TBI + Cy + etoposide) as previously described [7, 11, 12]. Conditioning selection was based on disease type and status at transplantation. Generally, patients with acute myeloid leukemia in complete remission (CR) were given BuCY, those with acute lymphoblastic leukemia in CR were given TBI + CY, and those in non-CR were administered the intensified regimen. In addition, some high-risk patients received the intensified regimen [7, 11, 12].

Cyclosporin A (CsA) plus methotrexate (MTX) (on days + 1, + 3, + 6) was administered to patients who underwent a matched sibling donor (MSD) transplant for GVHD prophylaxis. CsA + MTX + thymoglobulin (ATG; Genzyme, Cambridge) was used for patients who underwent a matched unrelated donor (MUD) transplant for GVHD. The CsA + MTX + ATG + mycophenolate (MMF) combination was administered to patients who underwent haploidentical donor (HID) transplant [7, 11, 12].

### Infection prophylaxis and treatment

At our institutions, oral sulfamethoxazole and norfloxacin were administered to all patients for infection prophylaxis [11, 12]. Ganciclovir was administered for the prophylaxis or treatment of cytomegalovirus (CMV) infection, whereas acyclovir was used for other viruses. Antifungal agents were prescribed for prophylaxis against fungal infections. Fluconazole (0.3 g/day) or itraconazole (0.4 g/kg/day) was used until 60 days posttransplantation for patients with no history of invasive fungal infection (IFI), while those with a history of IFI were given voriconazole (0.4 g/day), itraconazole (0.4 g/day), caspofungin (50 mg/day), or ambisome (0.5–1 mg/kg/day) intravenously. Oral voriconazole or itraconazole was prescribed as a substitute for intravenous (i.v) treatment when the peripheral white blood cell count was greater than 2.0 × 10^9/L and discontinued after 90 days posttransplantation.

Among antibiotics, carbapenems (imipenem or meropenem) combined with amikacin were administered as first-line antibiotics for patients developing fever during neutropenia. Vancomycin or piperacillin/tazobactam was used as the second-line antibiotic treatment. Other antibiotics were administered variably and to a minority of patients. For example, tigecycline was occasionally used for bacterial infections caused after administration of noneffective second-line antibiotics.

### Sequencing of 16S rRNA for fecal specimens

DNA from each stool specimen was extracted and purified, and the 16S rRNA gene between the V3 and V4 regions was amplified using PCR with modified universal bacterial primers, as described in our previous reports [7]. Microbiome DNA concentrations were detected using a qPCR assay, and sequencing was performed using the HiSeq2500 PE250 platform [7]. Sequencing data were screened and filtered according to quality and then aligned to the full-length 16S rRNA gene using the SILVA reference alignment as a template. Sequences were assembled into operational taxonomic units (OTUs) with 97% similarity [7].

### Microbiota analysis

Intestinal microbiota diversity was evaluated using the inverse Simpson index, as well as the number of operational taxonomic units (OTUs) and Shannon index as previously described [4, 7, 13]. Linear discriminant analysis (LDA) effect size (LEfSe) was applied to identify different microbiota characteristics using LEfSe software; the threshold on the logarithmic LDA score for discriminative features was 2.0 [7]. Phylogenetic classification at the family level was calculated according to a naive Bayesian classification scheme and the Greengenes reference database [4, 7, 14]. In addition, a nonparametric test (Mann–Whitney) was performed to compare the statistical differences between groups.

### Clinical metadata and survival prediction

All clinical data, including neutrophil recovery, were collected via retrospective review of clinical characteristics by individuals blinded to the microbiota of the participants. Neutrophil recovery was defined as an absolute neutrophil count was greater than 0.5 × 10^9/L posttransplantation for three consecutive days.

Area under receiver operating characteristic (ROC) curves (AUCs) from logistic regression analysis were used to evaluate the predictive performance of outcomes. The cutoff values of the inverse Simpson index and the selected bacteria were determined through AUC analysis of ROC curves for the predictive survival model by identifying the highest AUC, with a corresponding sensitivity and specificity. Based on the cutoff values for survival, a score of 0 or 1 was assigned for the inverse Simpson index and abundance of each microbial taxon (including Lachnospiraceae, Ruminococcaceae, Erysipelotrichaceae, and Enterobacteriaceae), where 0 represented a positive association with survival and 1 indicated a negative association. An accumulated intestinal microbiota (AIM) score was then generated by summing all the values (Table 1) [14–16]. Finally, the AIM score for predicting outcomes was determined by estimating the performance of the microbiota. The primary outcomes overall survival (OS) and disease-free survival (DFS) were analyzed; additionally, transplant-related mortality (TRM) was calculated.

**Table 1:** Predictive microbiota score for survival

Scoring system (relative abundance)	Score
Diversity (A)	
≥ 2.805	0
< 2.805	1
Lachnospiraceae (B)	
≥ 1.280%	0
< 1.280%	1
Ruminococcaceae (C)	
≥ 0.005%	0
< 0.005%	1
Erysipelotrichaceae (D)	
≥ 0.026%	0
< 0.026%	1
Enterobacteriaceae (E)	
≥ 27.602%	1
< 27.602%	0
AIM score*	
High	4–5
Low	0–3

^*^Accumulated intestinal microbiota (AIM) score: A + B + C + D + E; diversity, reverse Simpson index

### Statistical analysis

Data are summarized as median or mean ± SD for continuous variables. Categorical variables were compared using Pearson’s *χ*^2^ test or Fisher’s exact test. Correlations between the intestinal microbiota and groups were analyzed by “heatmap” estimation. The cumulative survival was analyzed using the Kaplan–Meier method and compared with the log-rank test (Mantel–Haenszel). Considering the competing risks of death and relapse, cumulative incidence curves in a competing risk setting were generated to calculate the probabilities of TRM, acute GVHD (aGVHD), and chronic (cGVHD) using the Gray test [11]. The Cox proportional hazards model was used to assess survival in multivariate analysis. Variables that were associated with survival (*P* < 0.20) in univariate analysis were included in the final Cox model. All *P*-values were considered two sided with a significance level of 0.05. SPSS 22.0 (SPSS, Chicago), or R software (version 3.1.1) was used to analyze all data [17], and *P* < 0.05 was defined as statistically significant.

## Results

### Clinical characteristics and bacterial selection

**Fig. 1:**
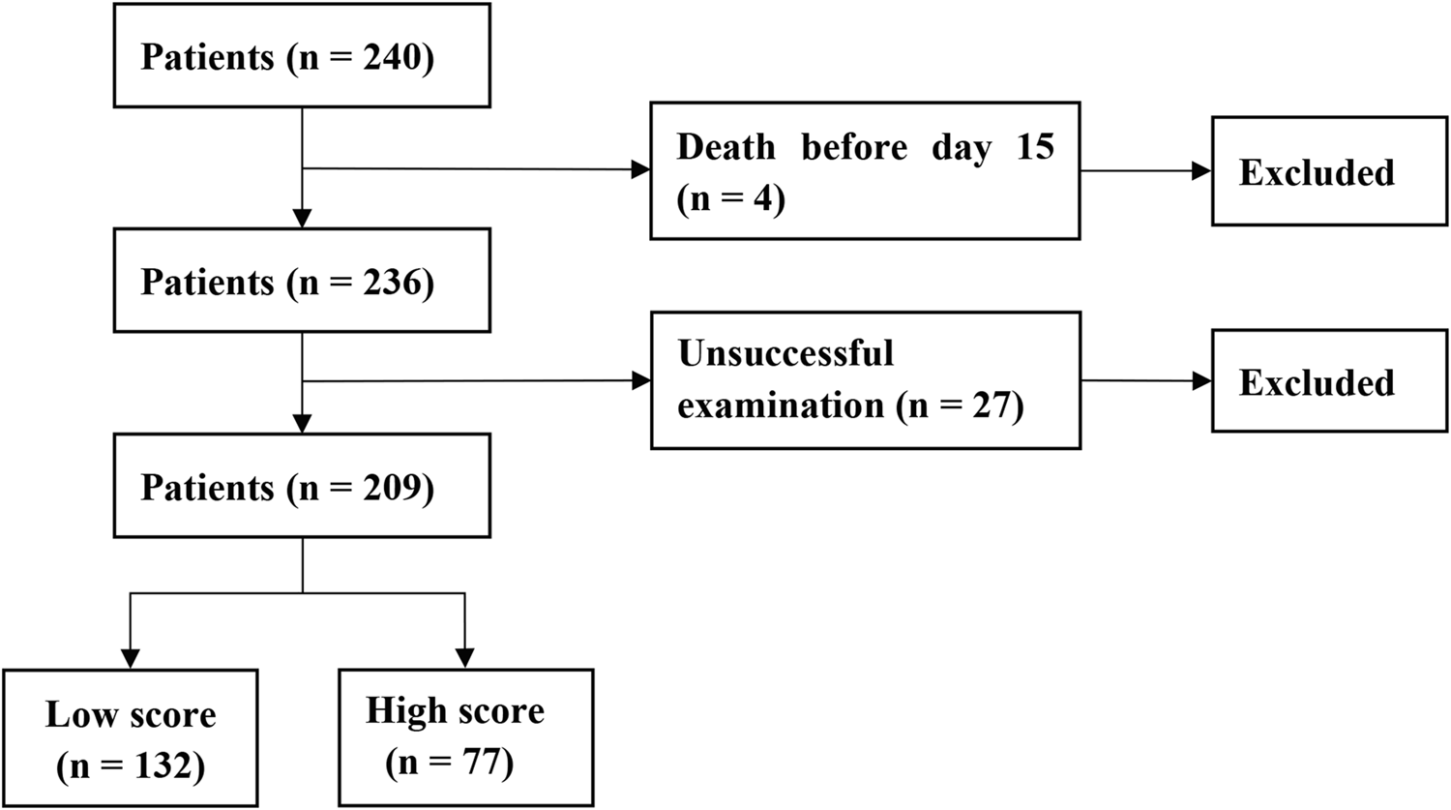
Diagram of patients enrolled in this study

**Fig. 2:**
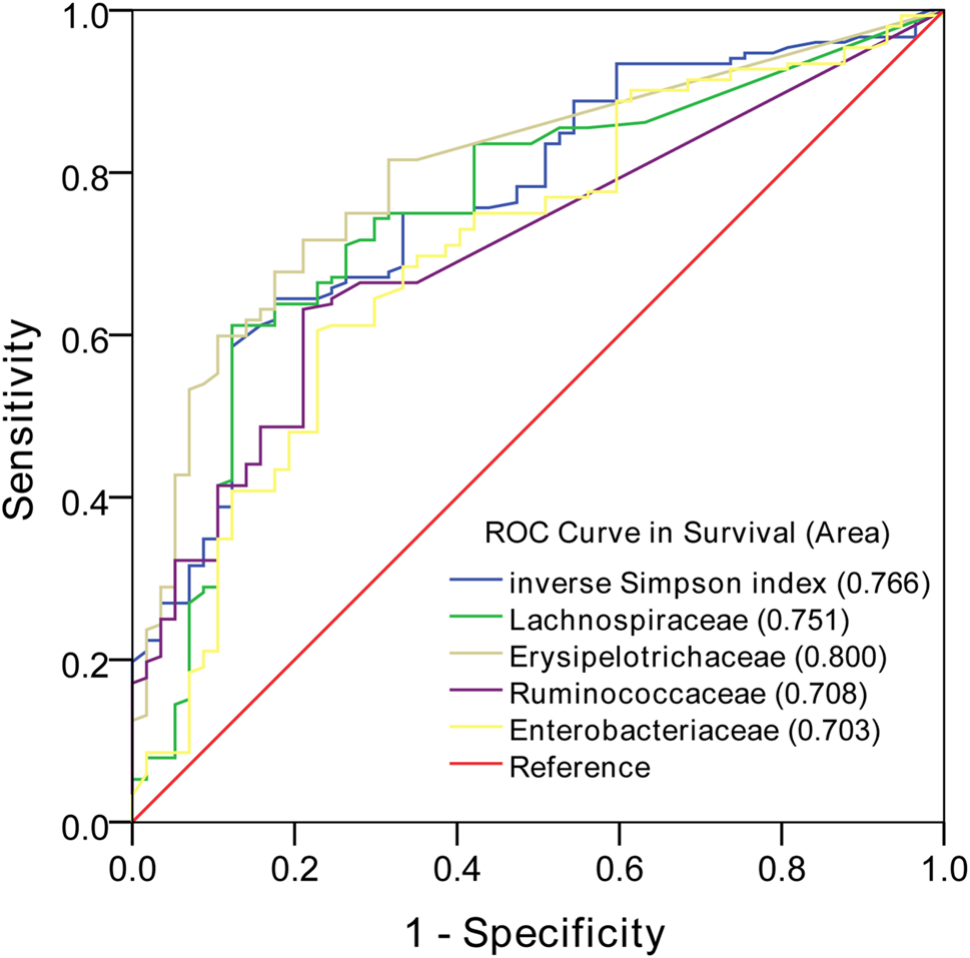
Microbiota diversity and the four bacterial taxa that predict survival were selected. ROC curves of the diversity and different bacteria at day 15 posttransplantation for the prediction of survival. ROC curve, area under receiver operating characteristic curve, diversity, reverse Simpson index

From the original cohort of 240 patients, 31 patients were excluded due to death before sample collection (*n* = 4) and unsuccessful fecal examination (*n* = 27) (Fig. 1). A total of 209 patients from the cohort were included in this study. We selected bacteria that had a significant negative or positive correlation with survival, as previously mentioned. Through AUC analysis of ROC plots for survival, the inverse Simpson index and abundance of four bacterial families, including Lachnospiraceae, Ruminococcaceae, Erysipelotrichaceae, and Enterobacteriaceae, were determined (AUC ≥ 0.70 and *P* < 0.01), and others showing an AUC < 0.70 or *P* ≥ 0.01 were excluded. The results demonstrated that the AUCs of diversity and abundance of the four aforementioned bacterial families were 0.766, 0.751, 0.708, 0.800, and 0.703, with sensitivity and specificity of 0.615 and 0.774, 0.590 and 0.849, 0.615 and 0.774, 0.686 and 0.736, and 0.609 and 0.811 for OS, respectively (Fig. 2). Based on cutoff values for survival, an AIM score was then generated from the assigned score for the diversity and abundance of the four bacterial families by adding all values (Table 1). The subjects were divided into low-score (0–3; *n* = 132) and high-score (4–5; *n* = 77) groups based on the AIM score (Tables 1 and 2).

**Table 2:** Patient and transplant characteristics for the groups

Variable	AIM score*		*P*
	Low (*n* = 132)	High (*n* = 77)	
Gender (%)			
Female	50 (37.9)	28 (36.4)	0.883
Male	82 (62.1)	49 (63.6)	
Age, years (%)			
< 32	65 (49.2)	35 (45.5)	0.667
> = 32	67 (50.8)	42 (54.5)	
Donor gender (%)			
Female	30 (22.7)	25 (32.5)	0.144
Male	102 (77.3)	52 (67.5)	
HLA mismatched (%)			
4–5	41 (31.1)	35 (45.5)	0.017
2–3	16 (12.1)	14 (18.2)	
0–1	75 (56.8)	28 (36.4)	
Underlying disease (%)			
ALL	44 (33.3)	27 (35.1)	0.939
AML	78 (59.1)	45 (58.4)	
MDS	10 (7.6)	5 (6.5)	
Genetics (%)			
Poor risk	78 (59.1)	46 (59.7)	0.927
Others	54 (40.9)	31 (40.3)	
Disease status at transplantation (%)			
CR	110 (83.3)	54 (70.1)	0.025
Non-CR	22 (16.7)	23 (29.9)	
Conditioning (%)			
Standard	104 (78.8)	42 (54.5)	< 0.001
Intensified	28 (21.2)	35 (45.5)	
Graft source (%)			
PBSC	98 (65.9)	52 (71.4)	0.445
BM + PBSC	34 (34.1)	25 (28.6)	
Bloodstream infection (%)	23 (17.4)	15 (19.5)	0.714
β-lactam (%)	76 (53.5)	58 (75.3)	0.002
Carbapenems	67 (50.8)	53 (68.8)	0.014
Piperacillin/Tazobactam	16 (12.1)	21 (27.3)	0.008
Cephalosporin	14 (10.6)	11 (14.3)	0.508
Vancomycin (i.v.) (%)	40 (30.3)	36 (46.8)	0.017
Amikacin (%)	44 (33.3)	28 (36.4)	0.654
III-IV aGVHD	8 (6.1)	25 (32.5)	< .001

*aGVHD*, acute graft-versus-host disease; *AIM score*, accumulated intestinal microbiota score; *ALL*, acute lymphoblastic leukemia; *AML*, acute myelogenous leukemia; *BM*, bone marrow; *CR*, complete remission; *i.v.*, intravenous; *MDS*, myelodysplastic syndrome; *PBSCs*, peripheral blood stem cells

The baseline characteristics of the patients are summarized in Table 2. Most of the transplant characteristics were similar between the two groups, such as donor gender, underlying disease and poor-risk genetics, graft source, and bloodstream infection. However, the high-score group included more patients who received an HLA mismatched (4/10–5/10 mismatched) donor transplant, *β*-lactam antibiotics (including carbapenems and piperacillin/tazobactam), or vancomycin (i.v.) than the low-score group (*P* = 0.017, 0.002, and 0.017, respectively); additionally, the number of patients with a non-CR status at transplant and who underwent intensified conditioning was higher in the high-score group than that in the low-score group (*P* = 0.025 and < 0.001, respectively; Table 2). No other variables were found to be significantly different between the groups (*P* > 0.05).

### Intestinal microbiota characteristics at neutrophil recovery

**Fig. 3:**
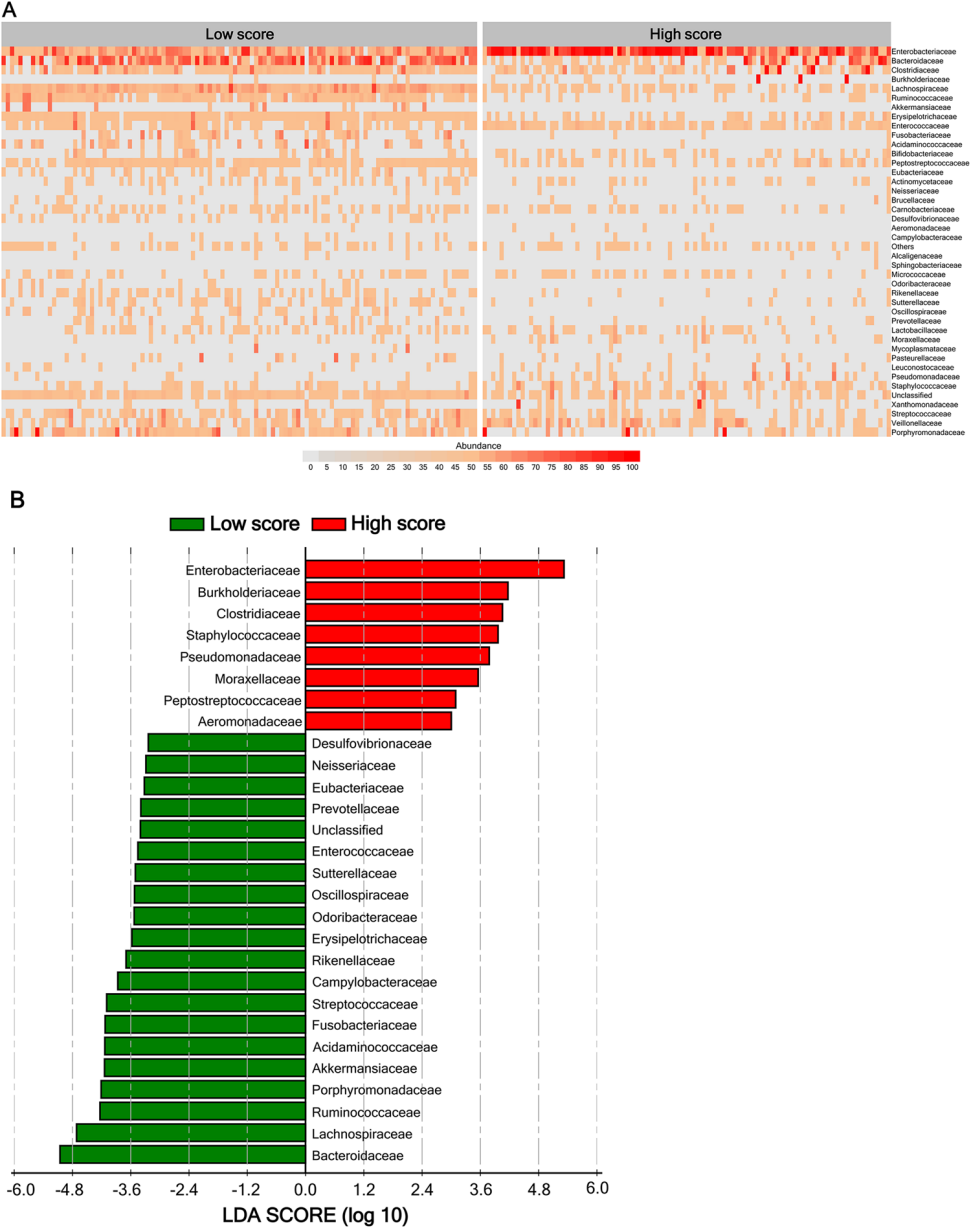
Differences of microbiota abundance and constitution at day 15 posttransplantation between the groups. **A** Heatmap of bacterial abundance in each group of patients. **B** Linear discriminant analysis (LDA) effect size (LEfSe) algorithm was used to identify the enriched bacterial family in each group

Although no difference was found between the low- and high-score groups before transplantation (pr-conditioning) (OTUs, 61 vs. 62; Shannon index, 1.91 vs. 1.89; *P* = 0.935 and 0.780, respectively, Fig. S1A and B), the number of OTUs and the Shannon index of microbiota diversity were higher in the low-score group than that in the high-score group (44.0 vs. 22.0, 1.83 vs. 0.66, respectively, both *P* < 0.001, Fig. S2A and B). To investigate enrichment differences in microbiota between the two groups, heatmap and LEfSe analyses were performed. We focused on the abundant bacterial families and compared their relative abundances between the two groups. The heatmap shows the abundance and phylogenetic composition of each subject in the two groups (Fig. 3A). In Fig. 3A, the transition from gray to red indicates the increase in microbiota abundance. The microbial abundance in the stool was higher in the low-score group than that in the high-score group (Fig. 3A). LEfSe analysis demonstrated that the enriched bacteria were significantly different between the groups (Fig. 3B). Furthermore, Lachnospiraceae, Ruminococcaceae, Erysipelotrichaceae, Bacteroidaceae, Akkermansiaceae, and Streptococcaceae were enriched in the low-score group, whereas Enterobacteriaceae, Aeromonadaceae, Peptostreptococcaceae, Moraxellaceae, Pseudomonadaceae, Staphylococcaceae, Clostridiaceae, and Burkholderiaceae were enriched in the high-score group (Fig. 3B).

As expected, other bacteria were different between the low- and high-score groups, for example, Bacteroidaceae and Porphyromonadaceae (38.846 vs. 0.006%, 1.135 vs. 0.000%, respectively, all *P* < 0.001), consistent with the results of LEfSe analysis, as shown in Fig. 3B. Taken together, patients with low and high scores showed significantly distinct intestinal microbiota.

We also explored the correlations between the single parameters of the AIM score (Fig. S3). The reverse Simpson index was positively associated with the abundance of Lachnospiraceae (*r* = 0.60, *P* < 0.001) and negatively associated with the abundance of Enterobacteriaceae (*r* =  − 0.42, *P* < 0.001). The abundance of Enterobacteriaceae was negatively correlated with that of Lachnospiraceae and Ruminococcaceae (*r* =  − 0.32 and − 0.26, respectively, both *P* < 0.001, Fig. S3).

#### GVHD

The median time of aGVHD emergence was day 21 (10–64) posttransplantation. The overall cumulative incidences of grades II-IV aGVHD by day + 100 posttransplant were 33.3% (29.2–37.4%) and 63.6% (58.1–69.1%) for the low- and high-score groups, respectively (*P* < 0.001, Fig. S4A). The cumulative incidences of grade III-IV aGVHD were 6.1% (4.1–8.2%) and 32.5% (27.2–37.8%) for the low- and high-score groups, respectively (*P* < 0.001, Fig. S4B). With regard to the correlation between AIM score and aGVHD severity, Spearman’s correlation analysis revealed a positive correlation (*r* = 0.397, *P* < 0.001). As for single-organ manifestations, of the 93 patients with grades II-IV aGVHD, 79, 72, and 31 experienced skin, intestinal, and hepatic aGVHD symptoms, respectively, and these organ manifestations were also correlated with the AIM score (*r* = 0.243, 0.487, and 0.345, respectively, all *P* < 0.001).

For the low- and high-score groups, the overall 3-year cumulative incidence of cGVHD posttransplantation was 38.8% (34.3–43.3%) and 30.0% (23.5–36.5%), respectively (*P* = 0.709, Fig. S4C), and that of extensive cGVHD was 11.9% (9.0–14.8%) and 11.4% (7.3–15.5%), respectively (*P* = 0.843, Fig. S4D).

### Survival and TRM

**Fig. 4:**
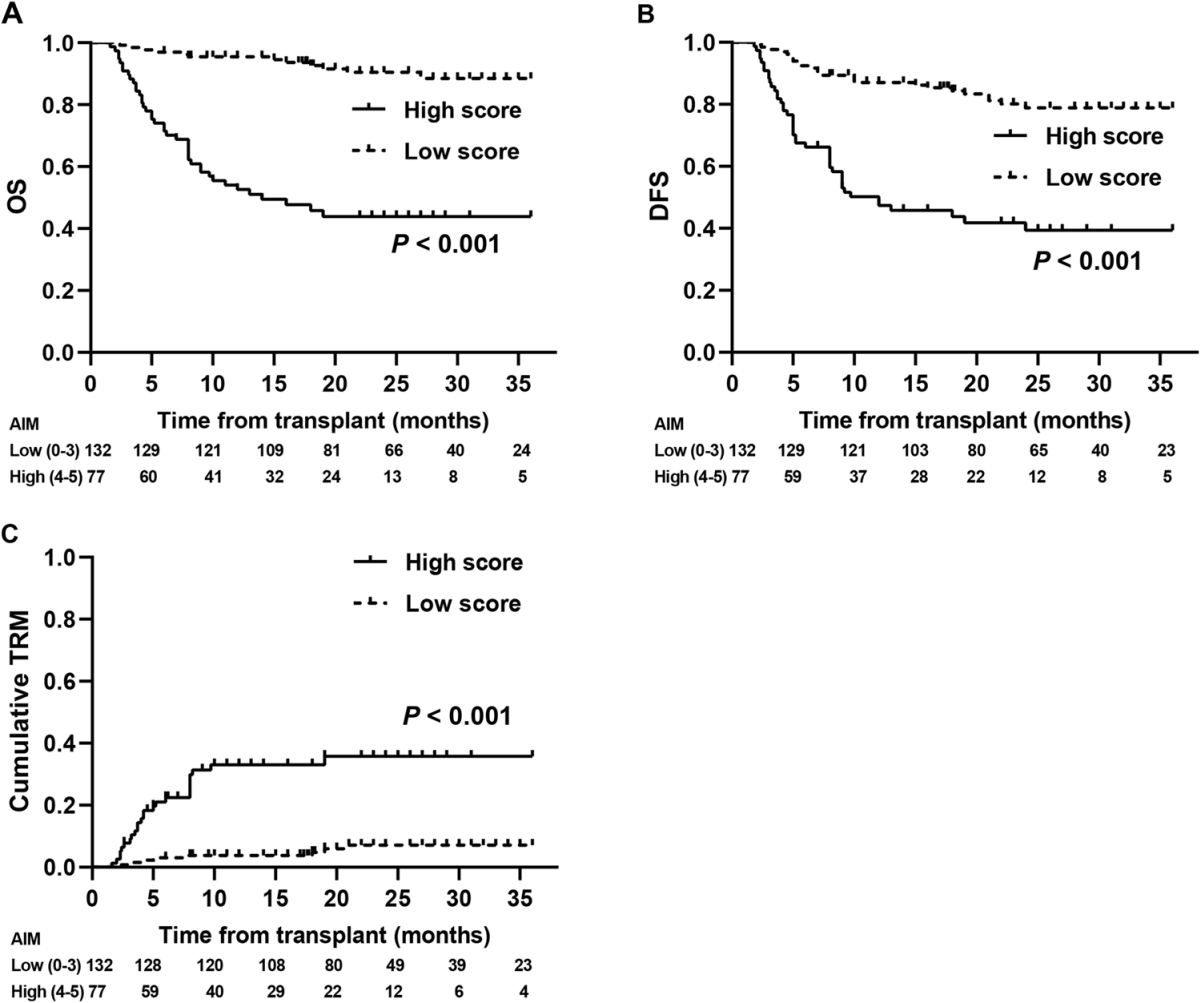
The accumulated intestinal microbiota (AIM) score and its association with survival. Both the 3-year cumulative overall survival (OS, **A**) and the disease-free survival (DFS, **B**) were longer in the low-score than those in the high-score group. **C** The 3-year cumulative transplant-related mortality (TRM) was considerably lower in the low-score group than that in the high-score group

The median follow-up was 19.0 months (range: 1.9–36 months). Based solely on the reverse Simpson index and the abundance of the four bacterial families, the 3-year cumulative OS was significantly different between each group with high and low index (or abundance) (all *P* < 0.001, Fig. S5A-E). The 3-year cumulative OS based on the AIM score posttransplantation was 88.5% (85.2–91.8%) and 43.9% (37.9–49.9%) for the low- and high-score groups, respectively (*P* < 0.001, Fig. 4A). The 3-year cumulative DFS posttransplantation was 78.9% (75.1–82.7%) and 39.3% (33.2–45.4%) for the low- and high-score groups, respectively (*P* < 0.001, Fig. 4B).

A total of 53 patients died at a median of 7.0 months (range: 1.6–19.0 months) during follow-up, and the causes of death included TRM (*n* = 33) and relapse (*n* = 20). Of the 33 patients who died of TRM, infection (*n* = 16, including 1 Epstein-Barr virus-associated posttransplant lymphoproliferative disorder) was the main cause of TRM. Other causes of death included aGVHD (*n* = 7), cGVHD (*n* = 3), intracranial hemorrhage (*n* = 1), hepatic veno-occlusive disease (*n* = 1), hemorrhagic cystitis (*n* = 1), thrombotic microangiopathy (*n* = 1), and multiple organ failure (*n* = 2), but the cause of death of 1 patient was unknown. The distribution of reasons for death between the two groups is shown in Table S1. The cumulative TRM is shown in Fig. 4C. The 3-year cumulative TRM posttransplantation was 7.1% (4.6–9.6%) and 35.8% (29.8–41.8%) for the low- and high-score groups, respectively *(P* < 0.001, Fig. 4C).

### Risk factors for survival and TRM

In multivariate analysis (Table 3), III-IV aGVHD, high AIM score, poor risk, and non-CR status during transplantation were independent risk factors for OS (hazard ratio [*HR*] = 4.95, 5.68, 1.62, and 1.84; 95% confidence interval [*CI*]: 2.60–9.42, 2.75–11.71, 1.14–2.31, and 1.00–3.38; *P* < 0.001, < 0.001, = 0.007, and 0.048, respectively). No other factors, including patient age, female donor, 4/10–5/10 HLA-mismatched donor, *β*-lactam administration, or conditioning intensity, were significantly associated with OS in multivariate analysis (all *P* > 0.05), although female donors, HLA-mismatched donors, and intensified conditioning were risk factors in univariate analysis (*P* = 0.015, 0.019, and 0.008, respectively). For DFS, poor-risk, non-CR status during transplantation, grades III-IV aGVHD, and high AIM scores were the independent risk factors (*HR* = 1.57, 2.23, 3.12, and 3.04; 95% *CI*: 1.18–2.10, 1.31–3.81, 1.74–5.60, and 1.74–5.31; *P* = 0.002, 0.003, < 0.001, and < 0.001, respectively). For TRM, III-IV aGVHD and high AIM scores were the independent risk factors (*HR* = 7.60 and 3.92; 95% *CI*: 3.43–16.83 and 1.57–9.79; *P* < 0.001 and = 0.003, respectively). Female donor, intensified conditioning, blood stream infection, and *β*-lactam administration were not identified as independent risk factors (all *P* > 0.05), although they were associated with TRM in univariate analysis (*P* = 0.002, 0.002, 0.015, and 0.009, respectively). Meanwhile, patient age, genetic risk, HLA mismatch, graft source, and administration of vancomycin were not associated with TRM (all *P* > 0.05).

**Table 3:** Multivariate analysis of outcomes

Factors	TRM		OS		DFS	
	Univariate	Multivariate (*P* (HR, 95% *CI*))	Univariate	Multivariate (*P* (HR, 95% *CI*))	Univariate	Multivariate (*P* (HR, 95% *CI*))
Gender						
Female	0.652	-	0.659	-	0.463	-
Male						
Patient age, years						
< 32	0.831	-	0.545	-	0.831	-
≥ 32						
Donor gender						
Female	0.002	0.100 (2.09, 0.87–5.05)	0.015	0.061 (1.87, 0.97–3.58)	0.226	-
Male						
Genetics						
Poor-risk	0.071	0.242 (1.30, 0.84–2.00)	0.003	0.007 (1.62, 1.14–2.31)	0.005	0.002 (1.57, 1.18–2.10)
Others						
Disease status						
Non-CR	0.138	0.439 (1.40, 0.59–3.32)	0.001	0.048 (1.84, 1.00–3.38)	< .001	0.003 (2.23, 1.31–3.81)
CR						
HLA mismatched						
4–5	0.216	-	0.019	0.315 (1.17, 0.86–1.61)	0.080	0.495 (1.10, 0.84–1.44)
2–3						
0–1						
Graft source						
PBSC	0.383	-	0.303	-	0.555	-
BM + PBSC						
Conditioning						
Standard	0.002	0.236 (1.64, 0.72–3.69)	0.008	0.841 (1.06, 0.58–1.94)	0.069	0.679 (1.13, 0.65–1.96)
Intensified						
Blood stream infection	0.015	0.315 (1.59, 0.64–3.94)	0.282	-	0.309	-
β-lactam	0.009	0.484 (1.86, 0.33–10.59)	0.098	0.468 (1.53, 0.48–4.87)	0.054	0.774 (1.18, 0.39–3.56)
Carbapenems	0.106	0.533 (1.53, 0.40–5.81)	0.191	0.561 (1.36, 0.48–3.85)	0.097	0.429 (1.49, 0.55–4.04)
Piperacillin/tazobactam	0.113	0.830 (1.10, 0.45–2.73)	0.136	0.615 (1.20, 0.60–2.40)	0.302	-
Cephalosporin	0.097	0.613 (1.42, 0.37–5.49)	0.490	-	0.153	0.818 (1.10, 0.48–2.55)
Vancomycin (i.v.)	0.480	-	0.936	-	0.883	-
Amikacin	0.232	-	0.851	-	0.643	-
III-IV aGVHD	< .001	< .001 (7.60, 3.43–16.83)	< .001	< .001 (4.95, 2.60–9.42)	< .001	< .001 (3.12, 1.74–5.60)
AIM score*						
High (4–5)	< .001	.003 (3.92, 1.57–9.79)	< .001	< .001 (5.68, 2.75–11.71)	< .001	< .001 (3.04, 1.74–5.31)
Low (0–3)						

*aGVHD*, acute graft-versus-host disease; *AIM score*, accumulated intestinal microbiota score; *BM*, bone marrow; *CI*, confidence interval; *CR*, complete remission; *DFS*, disease-free survival; *HR*, hazard ratio; *i.v.*, intravenous; *OS*, overall survival; *PBSC*, peripheral blood stem cell; *TRM*, transplant-related mortality

### Microbiota survival prediction

**Fig. 5:**
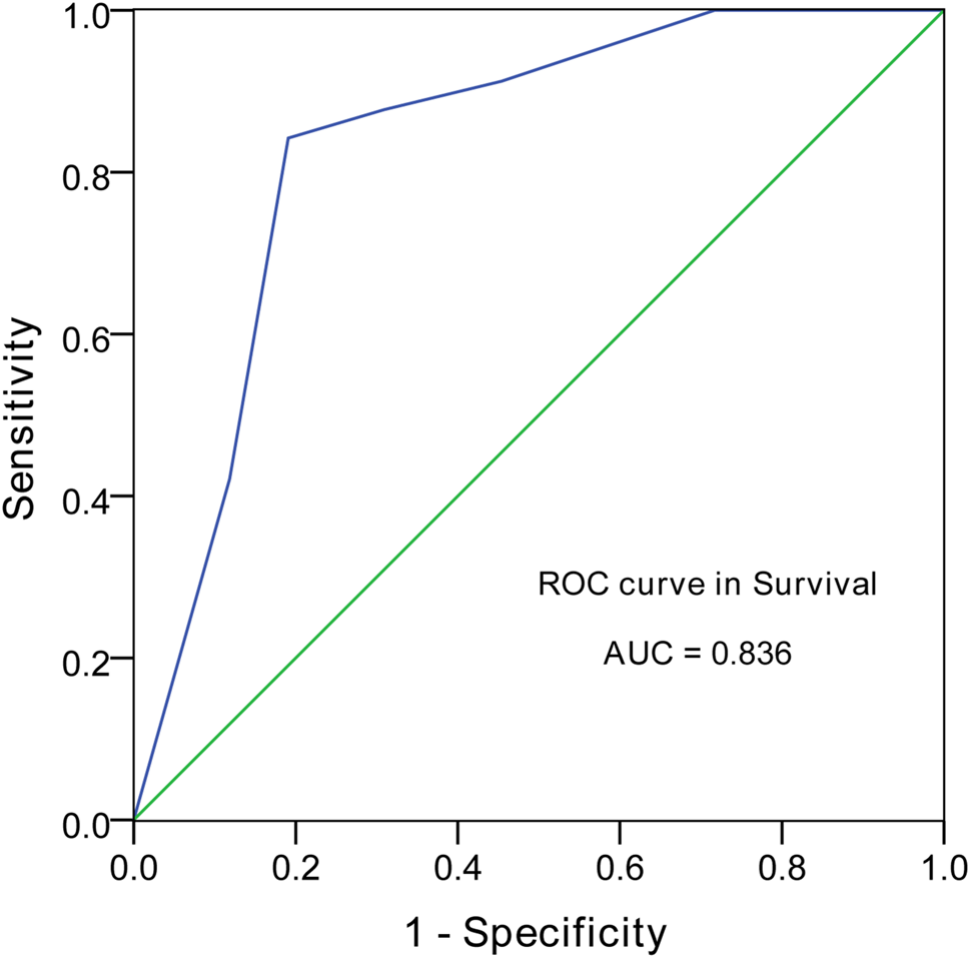
The accumulated intestinal microbiota (AIM) score predicts survival. AIM score, accumulated intestinal microbiota score; AUC, area under receiver operating characteristic curve; ROC, receiver operating characteristic curve

To better explore the potential clinical effects of the microbiota markers, the inverse Simpson index and the abundance of Lachnospiraceae, Ruminococcaceae, Erysipelotrichaceae, and Enterobacteriaceae were scored based on the cutoff values for survival. Subsequently, the AIM score for survival was calculated. The result of the ROC curve analysis for the predictive model indicated that the AIM score could serve as a predictor for survival (*AUC* = 0.836, *P* < 0.001; cutoff value, 3.5), with a sensitivity and specificity of 0.842 and 0.809, respectively (Fig. 5). Furthermore, we compared the AUC of the AIM score with that of the five factors and found that the AUC of the AIM score was higher than that of the inverse Simpson index as well as Lachnospiraceae, Ruminococcaceae, and Enterobacteriaceae abundance (*P* = 0.004, < 0.001, < 0.001, < 0.001, respectively), although the AUC of Erysipelotrichaceae abundance was not significantly different from that of the AIM (*P* = 0.152). In addition, we could not identify that one factor was more important for the prediction than the other (*P* > 0.05).

## Discussion

The relationship between intestinal microbiota and survival has gained increasing attention in recent years [4, 18, 19]. Taur Y et al. [4] observed that a low intestinal microbiota diversity was positively correlated with worse survival. Peled JU [8] reported that profound microbiota injury, that is, loss of diversity and domination by a single taxon, was associated with mortality. In this study, we found that loss of Lachnospiraceae, Ruminococcaceae, and Erysipelotrichaceae in addition to diversity and a bloom of Enterobacteriaceae in patients undergoing allo-HSCT at neutrophil recovery was associated with poor survival, and that the cumulative AIM score from these four bacteria taxa and diversity could predict survival.

Previous studies have demonstrated that low microbiota diversity is associated with allo-HSCT complications, such as infections and aGVHD, which, at least in part, leads to poor survival [3, 8, 20, 21]. A few studies have indicated that intestinal microbiota could be a predictor of mortality during allo-HSCT [4, 8, 22]. However, studies on specific bacteria and their roles in predicting survival and mortality are inconsistent. A previous study indicated that Gammaproteobacteria is associated with mortality [4]. Recently, a study suggested that Enterococcus could be a predictor of ravaged microbiota and poor prognosis after allo-HSCT [23].

This study mainly focused on determining whether specific microbiota is a biomarker for survival. First, our results demonstrated that both the diversity and abundance of microbiota were positively (Lachnospiraceae) or negatively (Enterobacteriaceae) correlated with survival, and the microbiota score was associated with aGVHD occurrence. The associations between the microbiota and survival were consistent with those reported in our previous study on microbiota in aGVHD, although the prediction of survival from Peptostreptococcaceae was replaced by Ruminococcaceae due to a lower ROC area [15]. The findings for Lachnospiraceae, Ruminococcaceae, and Erysipelotrichaceae were in accordance with those reported by Peled JU, and a new finding of a bloom in Enterobacteriaceae that was associated with mortality, which might be attributed to vancomycin administration as described in our previous report [7, 8]. Second, we found that other bacteria, including Bacteroidaceae and Porphyromonadaceae, had a positive association, and Enterococcaceae had a negative association with survival, but the AUC of the latter in survival prediction was less than 0.70 (data not shown). In addition, we observed that the high-score group comprised more patients who received an HLA-mismatched (4/10–5/10 mismatched) donor transplant, and β-lactam antibiotics and vancomycin (i.v.), as well as patients who had a non-CR status at transplantation and underwent intensified conditioning than the low-score group; however, no difference in the microbiota diversity was identified between the groups before transplantation. These findings suggest that different antibiotics, conditioning, and disease status might have multiple effects on the microbiota [7, 15, 17, 24, 25].

Furthermore, this study indicated that microbiota diversity combined with the four bacterial families could serve as a potent predictor of survival. A lower AIM score was associated with better survival and lower transplant-related mortality. Our study indicates that the differentiation of survival based on the AIM score is superior to that of diversity alone. The bacteria of the AIM score in predicting survival were consistent with that in our previous study with regard to aGVHD prediction [15, 17]. Additionally, the intensity of microbiota disruption accompanied TRM at a certain level. The AIM score may be effective and convenient for clinical applications with high sensitivity and specificity. With regard to the mechanism of the association between the four bacteria and survival, studies have demonstrated that Lachnospiraceae, Ruminococcaceae, and Erysipelotrichaceae can maintain intestinal homeostasis, induce tolerance via their metabolites, and then decrease the occurrence of infection and serious aGVHD [3, 7, 26]. However, the bloom of Enterobacteriaceae is thought to be involved in injury of intestinal mucosa and homeostasis, resulting in lipopolysaccharide biosynthesis, promotion of occurrence of infectious diseases and GVHD, and an increase in mortality [3, 7, 26].

In multivariate analysis, a high AIM score, which corresponds to serious microbiota disruption, and III-IV aGVHD were independent risk factors for TRM. These results were consistent with those of a recent report in which patients with skewed microbiota displayed a high frequency of posttransplantation mortality [20]. With regard to the effects of the microbiota on the outcome of allo-HSCT, our findings are in accordance with previous studies that have reported mechanisms through which the microbiome modulates alloreactivity [9, 26–32]. A limitation of this study is the fact that it was a retrospective analysis, and the sample size was not relatively large; thus, our results should be validated in a larger prospective study.

## Conclusions

This study indicated that the intestinal microbiota score could predict survival following allo-HSCT. Our data may guide clinicians to determine the patients who are at serious risk of mortality. Furthermore, the results suggest that these patients should consider and implement interventions to restore the integrity of intestinal microbiota, such as fecal microbiota transplantation or preemptive treatment strategies. Future studies are needed for a more in-depth investigation of the mechanism underlying the microbiota disruption of outcomes; additionally, a larger prospective validation study from multiple centers is essential in the future.

## Supplementary Information

Below is the link to the electronic supplementary material.

Supplementary file1 (DOC 1777 KB)
