# Supplementary material for: Intestinal microbiota score could predict survival following allogeneic hematopoietic stem cell transplantation
Source: Ann Hematol. 2022 Mar 25;101(6):1283–94. doi: 10.1007/s00277-022-04817-8 (PMC9072276; doi:10.1007/s00277-022-04817-8)
Supplement: Supplementary file 1 — Supplementary file1 (DOC 1777 KB) [file 277_2022_4817_MOESM1_ESM.doc]

**Supplementary Information**

**Supplementary tables**

**Table S1** **Cause of death in the two groups**

| Cause of death |  | AIM score | | |
| --- | --- | --- | --- | --- |
|  |  | Low (n = 12) |  | High (n = 41) |
| Relapse |  | 4 |  | 16 |
| Infection |  | 3 |  | 13 |
| aGVHD |  | 2 |  | 5 |
| cGVHD |  | 1 |  | 2 |
| Intracranial hemorrhage |  | 0 |  | 1 |
| Hepatic veno-occlusive disease |  | 0 |  | 1 |
| Hemorrhagic cystitis |  | 0 |  | 1 |
| Thrombotic microangiopathy |  | 1 |  | 0 |
| Multiple organ failure |  | 1 |  | 1 |
| Unknown |  | 0 |  | 1 |

AIM score, accumulated intestinal microbiota score; aGVHD, acute graft-versus-host disease; cGVHD, chronic GVHD.

**Supplementary figures**


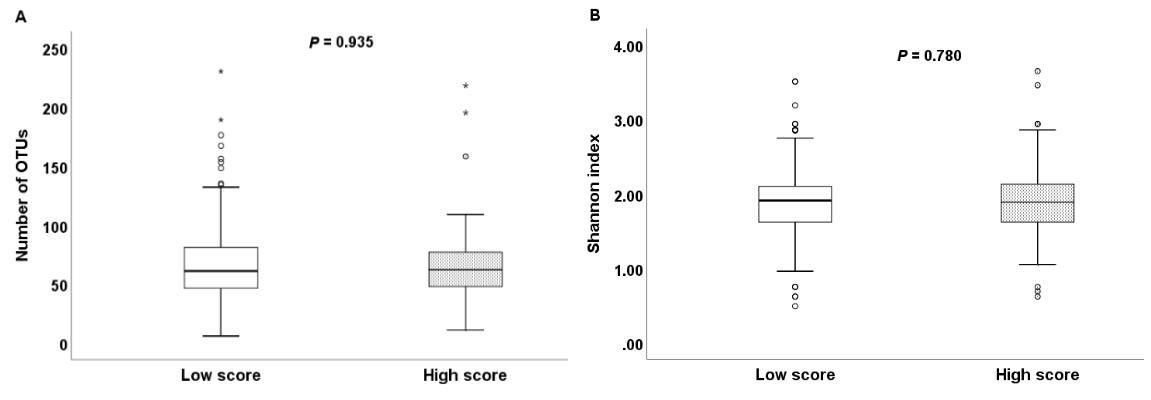


**Fig. S1 Microbiota diversity before transplantation (pre-conditioning) in the groups with different accumulated intestinal microbiota (AIM) scores**

No difference was found in the number of operational taxonomic units (OTUs, **A**) and Shannon index (**B**) between the low- and high-score groups (OTUs: 62 vs. 61, Shannon index: 1.89 vs. 1.91, *P* = 0.935, 0.780, respectively).


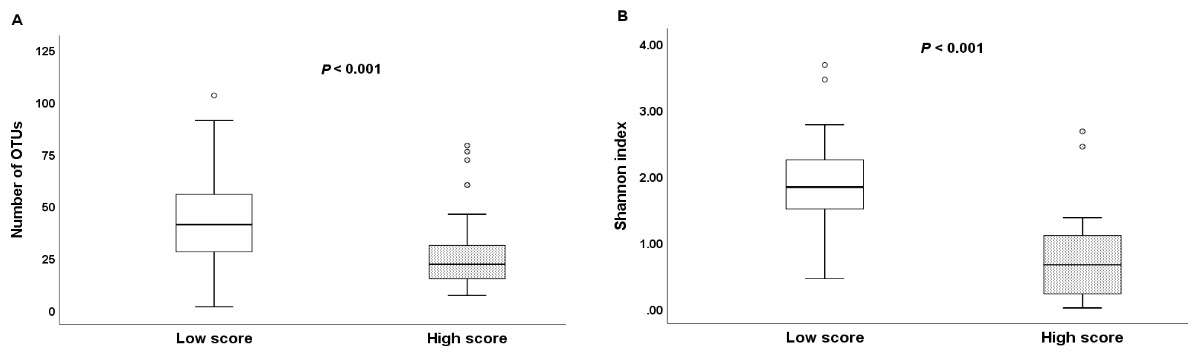


**Fig. S2 Comparison of microbiota diversity at day 15 ± 1 post-transplantation between groups with different accumulated intestinal microbiota (AIM) scores**

The number of operational taxonomic units (OTUs, A) and Shannon index (B) were calculated for the low- and high-score groups.


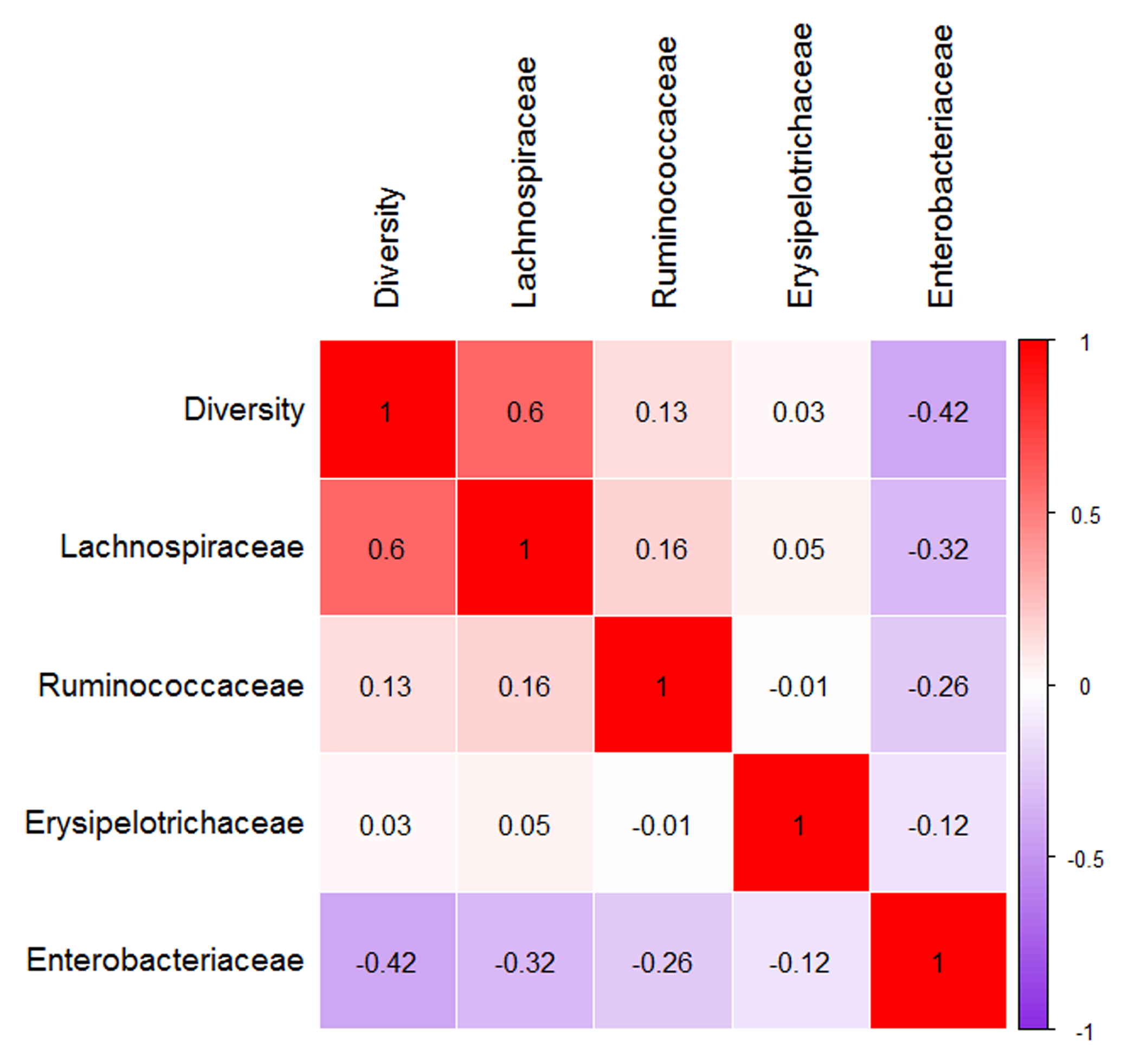


**Fig. S3 Correlations between the parameters of the accumulated intestinal microbiota (AIM) score**

Correlations between the parameters of the AIM score; the index of correlation coefficients is shown in each square. Red is representative of positive correlation, while purple is representative of negative correlation. Diversity, reverse Simpson index.


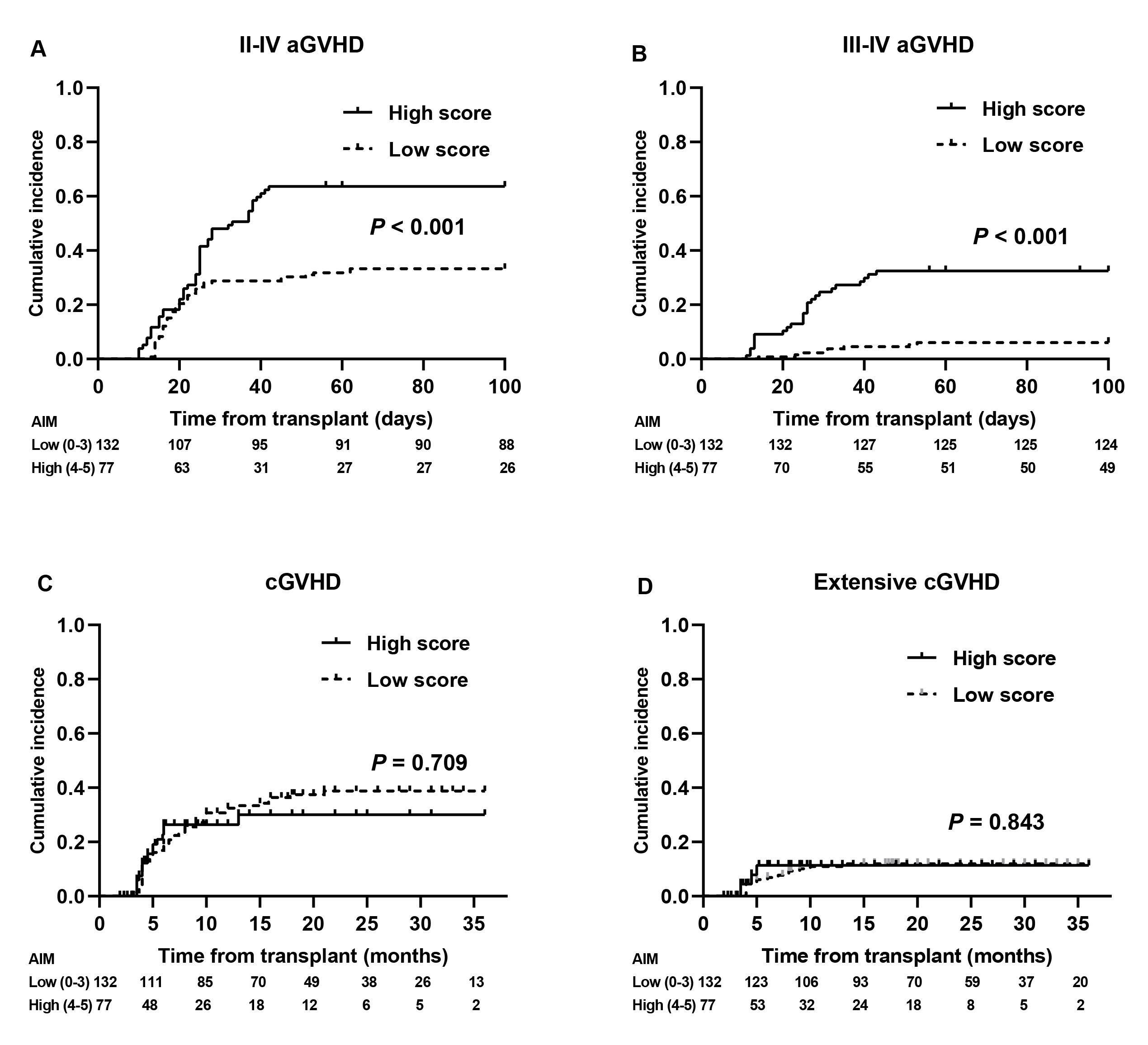


**Fig.S4 Cumulative incidence of GVHD according to the accumulated intestinal microbiota (AIM) score**

The overall cumulative incidences of grade II-IV acute graft-versus-host disease (aGVHD) (**A**) by day +100 post-transplant were 33.3% and 63.6% and grade III-IV aGVHD (**B**) were 6.1% and 32.5%, respectively, for the low- and high-score groups (all *P* < 0.001). The overall 3-year cumulative incidences of chronic GVHD (cGVHD) (**C**) post-transplantation were 38.8% (34.3-43.3%) and 30.0% (23.5-36.5%) and extensive cGVHD (**D**) were 11.9% (9.0-14.8%) and 11.4% (7.3-15.5%), respectively, for the low- and high-score groups (*P* = 0.709 and 0.843, respectively).


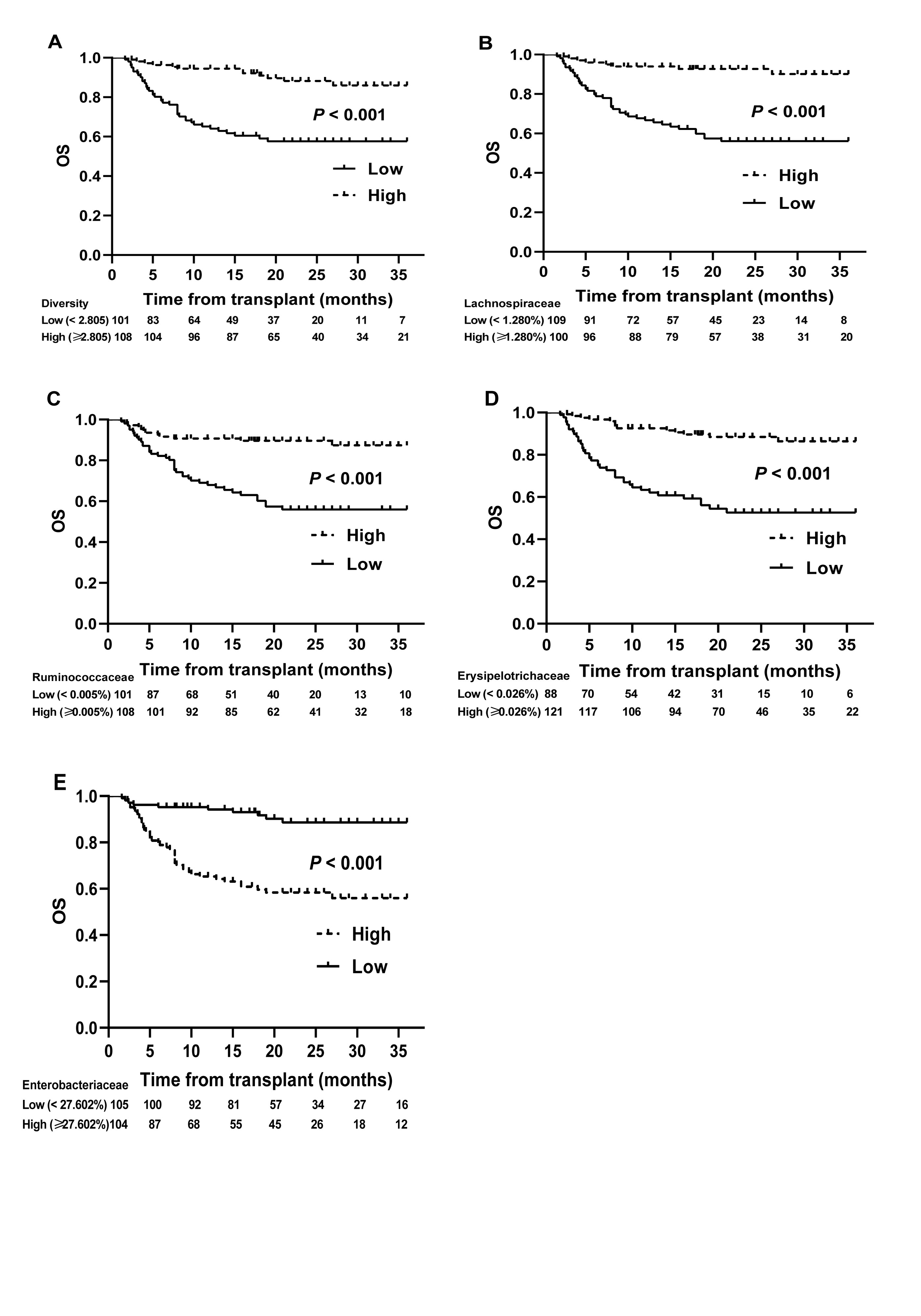


**Fig. S5 Cumulative overall survival (OS) according to each factor of the five parameters**

Based solely on the reverse Simpson index (diversity) and the abundance of the four bacterial taxa, the 3-year cumulative OS was significantly different between each group with high and low index (or abundance) (all *P* < 0.001).
